# Artesunate enhances adriamycin cytotoxicity by inhibiting glycolysis in adriamycin-resistant chronic myeloid leukemia K562/ADR cells

**DOI:** 10.1039/c8ra08041k

**Published:** 2019-01-09

**Authors:** Li Chen, Chao Wang, Ning Hu, Hongmian Zhao

**Affiliations:** Department of Hematology, Huaihe Hospital of Henan University No. 115 Ximen Street Kaifeng 475000 Henan China zhhm6039@163.com

## Abstract

Adriamycin (ADR) is a widely used drug in multiple cancers including leukemia. Artesunate (ART) has been reported to improve the cytotoxicity of some chemotherapeutic drugs in cancers. However, it is still unknown whether ART can augment the cytotoxicity of ADR in ADR-resistant K562 leukemia cells (K562/ADR). Glycolytic activity was assessed by detecting glucose consumption, lactate production and glycolysis-related enzymes. MDR1 and ABCG2 mRNA levels were measured by RT-qPCR. Protein levels of P-glycoprotein (P-gp), ABCG2 and cytochrome *c* (Cyt *c*) were determined by western blot assay. Cell apoptosis was evaluated by flow cytometry. Cell viability was examined by CCK-8 assay. The results showed that K562/ADR cells exhibited increased glycolytic activity and *mdr1* and *abcg2* gene expression. ART potentiated ADR cytotoxicity in K562 and K562/ADR cells. Moreover, ART reversed ADR-induced *mdr1* and *abcg2* gene expression and inhibited P-gp and ABCG2 activities in K562/ADR cells. ART potentiated ADR-mediated inhibition on glycolysis in K562 and K562/ADR cells. Inhibition of glycolysis reduced cell viability, downregulated the expression of *mdr1* and *abcg2* genes, and induced cell apoptosis in K562/ADR cells. Overall, the results indicated that ART enhanced ADR cytotoxicity by inhibiting glycolysis and reducing *mdr1* and *abcg2* gene expression in K562/ADR cells, providing a deep insight into the function and molecular basis of ART in regulating MDR in leukemia cells and hinting at the potential values of ART in alleviating MDR in cancers.

## Abstract 2

Adriamycin (ADR) is a widely used drug in multiple cancers including leukemia.

## Graphical abstract

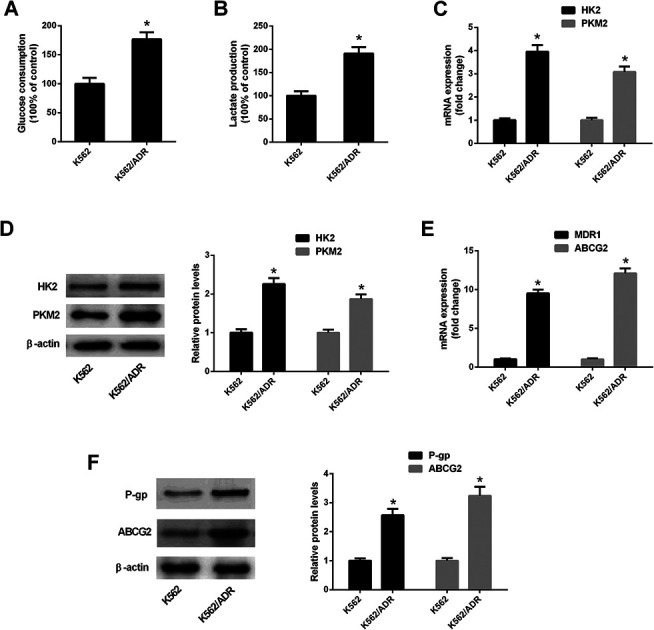

## 1. Introduction

Leukemia is the ninth most frequently diagnosed cancer in the United States with an estimated 60 300 new cases in 2018 alone, accounting for approximately 4% of all new cancer cases and cancer-related deaths.^1^ Leukemia can be classified into four major types: acute lymphoblastic leukemia (ALL), acute myeloid leukemia (AML), chronic lymphocytic leukemia (CLL), and chronic myeloid leukemia (CML).^1^ Although CML is the fourth most common type of leukemia, it remains a serious threat for human health and life with a median duration of 5–6 years if untreated.^2^ CML progresses from the chronic phase through the accelerated phase to blast crisis.^2^ Chemotherapy is the primary countermeasure for leukemia, whereas the existence and development of drug resistance especially multidrug resistance (MDR) is a massive clinical obstacle to effective chemotherapy for patients with leukemia including CML.^3,4^ In tumor cell lines, ATP-binding cassette (ABC) transporters such as P-glycoprotein (P-gp; encoded by *mdr1* gene) and ABCG2 function as crucial players in the maintenance and development of MDR resistance.^5,6^ The overexpression of P-gp and ABCG2 reduce the cytotoxicity of a broad spectrum of antitumor drugs such as anthracyclines, podophyllotoxins and taxanes.^7,8^

Adriamycin (ADR), an anthracycline antibiotic derived from *Streptomyces peucetius*, has been widely used to treat various cancers including leukemia.^9^ However, drug resistance of ADR strikingly limited its utilization in cancer management.^10,11^ Artesunate (ART), a semi-synthetic derivative of artemisinin extracted from *Artemisia annua* L. (Sweet wormwood), is the World Health Organization (WHO)-approved first-line treatment drug for malaria.^12^ ART has potential anti-tumor activities in multiple malignancies such as ovarian cancer,^13^ hepatocellular cancer^14^ and prostate cancer.^15^ Also, ART is a promising candidate for leukemia therapy due to its potential anti-leukemic activity in leukemic cell lines and murine models.^16–19^ ART could improve the cytotoxicity of some chemotherapeutic drugs in cancers. For instance, ART increased cytotoxicity conferred by cisplatin by downregulating RAD51 in ovarian cancer cells.^20^ ART enhanced the cytotoxicity of ADR and mitigated ADR resistance in esophageal cancer cells with ABCG2 overexpression.^21^

Glycolysis, the process of conversion of glucose into pyruvate followed by lactate productions, is the main energy producing pathway under hypoxic conditions.^22^ Glycolysis is markedly increased in tumor cells, and increased glycolysis favors the development of cancers and confers selective advantage to cancer cells under diminished nutrient supply.^23,24^ Enhanced glycolysis also has been shown to be closely linked with drug resistance.^25,26^ In the present study, we demonstrated that ART enhanced ADR cytotoxicity by inhibiting glycolysis and downregulating *mdr1* and *abcg2* gene expression in multidrug-resistant leukemia cells.

## 2. Materials and methods

### 2.1. Cell culture and reagents

Human CML cell line K562 and ADR-resistant CML cell line K562/ADR were purchased from the Cell Bank of Shanghai Institute of Biochemistry and Cell Biology (Shanghai, China). K562 and K562/ADR cells were cultured in RPMI 1640 medium (Gibco, Grand Island, NY, USA) supplemented with 10% fetal bovine serum (FBS; Gibco). To maintain drug resistance, 1 μg ml^−1^ ADR (Sigma-Aldrich, St. Louis, MO, USA) was added to the culture medium of K562/ADR cells. ART (purity >98%) and 2-deoxyglucose (2-DG) were purchased from Sigma-Aldrich.

### 2.2. Glucose consumption and lactate production determination

K562 or K562/ADR cells were seeded into 24-well plates at a density of 2 × 10^4^ cells per well. At 48 h after treatment, cell medium was collected and glucose concentration was determined using a glucose assay kit (Biovision, Mountain View, CA, USA) following the protocols of manufacturer. Lactate production was determined by a Lactate Assay kit (Biovision) according to the instructions of manufacturer.

### 2.3. Reverse transcription-quantitative polymerase chain reaction (RT-qPCR) assay

Total RNA was extracted from K562 or K562/ADR cells using Trizol reagent (Invitrogen, Carlsbad, CA, USA). cDNA first strand was then synthesized using M-MLV reverse transcriptase (Invitrogen) and random primers, followed by real time qPCR analysis using Fast SYBR Green Master Mix (Thermo Fisher Scientific) and specific quantitative primers on Applied Biosystems PRISM 7500 real time PCR detection system (Thermo Fisher Scientific). The primer sequences were as follows: 5′-AAGCTTAGTACCAAAGAGGCTCTG-3′ (forward) and 5′-GGCTAGAAACAATAGTGAAAACAA-3′ (reverse) for MDR1; 5′-GTCGAAGCCCCATAGTGAAG-3′ (forward) and 5′-GTGAATCAATGTCCAGGCGG-3′ (reverse) for pyruvate kinase 2 (PKM2); 5′-CCGCGACAGTTTCCAATGACCT-3′ (forward) and 5′-GCCGAAGAGCTGCTGAGAACTGTA-3′ (reverse) for ABCG2; 5′-ATTGTCCAGTGCATCGCGGA-3′ (forward) and 5′-AGGTCAAACTCCTCTCGCCG-3′ (reverse) for hexokinase 2 (HK2); 5′-TTCCAGCCTTCCTTCCTGGG-3′ (forward) and 5′-TTGCGCTCAGGAGGAGCAAT-3′ (reverse) for β-actin. β-actin acted as the internal control to normalize the expression of MDR1, PKM2, ABCG2 or HK2.

### 2.4. Western blot assay

Cells were lysed using RIPA Lysis and Extraction Buffer (Thermo Fisher Scientific) containing Protease Inhibitor Cocktail (Sigma-Aldrich). Protein in cell supernatant was quantified by Bradford protein assay (Bio-Rad Laboratories). Equal amount of protein (30 μg per lane) was separated by SDS-PAGE and transferred to nitrocellulose membranes (Millipore, Billerica, MA, USA). Subsequently, the membranes were blocked with 5% non fat milk and then incubated with primary antibody against HK2, PKM2, P-gp, ABCG2, cytochrome *c* (Cyt *c*), or β-actin overnight at 4 °C. Next, the membranes were probed with horseradish peroxidase (HRP)-labeled secondary antibody for 1 h at room temperature. Finally, immunoreactive signals were detected using enhanced chemiluminescence (ECL) reagent (Thermo Fisher Scientific) and quantified using Quantity One version 4.6.6 software (Bio-Rad Laboratories). β-actin functioned as the loading control. All antibodies were purchased from Abcam (Cambridge, UK).

### 2.5. Cell Counting Kit-8 (CCK-8) assay

Cell viability was determined by CCK-8 assay referring to the protocols of manufacturer. Briefly, cells were seeded into 96-well plates at a density of 10^5^ cells per well in 100 μl of culture medium and cultured overnight. ADR, ART or 2-DG with various appointed concentrations was added into 96-well plates. At 48 h after drug stimulation, 10 μl of CCK-8 solution was added into each well. The plates were then incubated for another 2 h in the incubator. The optical density values were recorded at 450 nm using a Thermo Scientific Microplate Reader (Thermo Fisher Scientific). Data from three replicates were presented as the percentage of treated cells relative to untreated controls.

### 2.6. Cell apoptosis detection

Cell apoptotic rate was determined using an Annexin V-FITC Apoptosis Detection Kit (Sigma-Aldrich) following the instructions of manufacturer. Briefly, cells were collected at 48 h post stimulation and resuspended in 1× binding buffer at a concentration of about 1 × 10^6^ cells per ml. Subsequently, 500 μl of cell suspension were co-incubated with 5 μl of Annexin V FITC Conjugate and 10 μl of Propidium Iodide Solution for 10 min in the dark. Finally, cell apoptosis was analyzed using a flow cytometer (BD Biosciences, San Jose, CA, USA). Caspase 3/7 activity was determined using Cell Meter™ Caspase 3/7 Activity Apoptosis Assay Kit (AAT Bioquest®, Inc., Sunnyvale, CA, USA) according to the protocols of manufacturer.

### 2.7. Evaluation of P-gp and ABCG2 activities

Activities of P-gp and ABCG2 were evaluated by a substrate accumulation assay. K562/ADR cells seeded in the 24-well plates at a density of 10^5^ cells per well. Cells were treated with 10 μM ART for 24 h, 50 μM verapamil (a positive control inhibitor of P-gp) for 0.5 h, or 10 μM Ko140 (a positive control inhibitor of ABCG2) for 0.5 h at 37 °C. Then, P-gp substrate rhodamine123 (R123; 5 μM) or ABCG2 substrate pheophorbide A (Phe A; 10 μM) was added and incubated for an additional 0.5 h. Cells were collected, rinsed twice with cold PBS, and lysed with 0.8% Triton X-100. Protein concentration of cell lysates was quantified by Bradford protein assay (Bio-Rad Laboratories). The fluorescence intensity of R123 or Phe A was measured using a SpectraMAX M5 Microplate Reader (Molecular Devices, Los Angeles, CA, USA) at an excitation wavelength of 485 nm and emission wavelength of 535 nm for R123, and an excitation wavelength of 635 nm and emission wavelength of 670 nm for Phe A.

### 2.8. Statistical analysis

All results from three independent experiments were shown as mean ± standard deviation. Student's *t*-test was used to perform two group comparisons and one-way analysis of variance (ANOVA) was employed for three group comparisons. Statistical analysis was conducted using SPSS 16.0 (SPSS, Inc., Chicago, IL, USA) with *P* < 0.05 as statistically significant.

## 3. Results

### 3.1. K562/ADR cells exhibited increased glycolytic activity and MDR gene expression

**Fig. 1:**
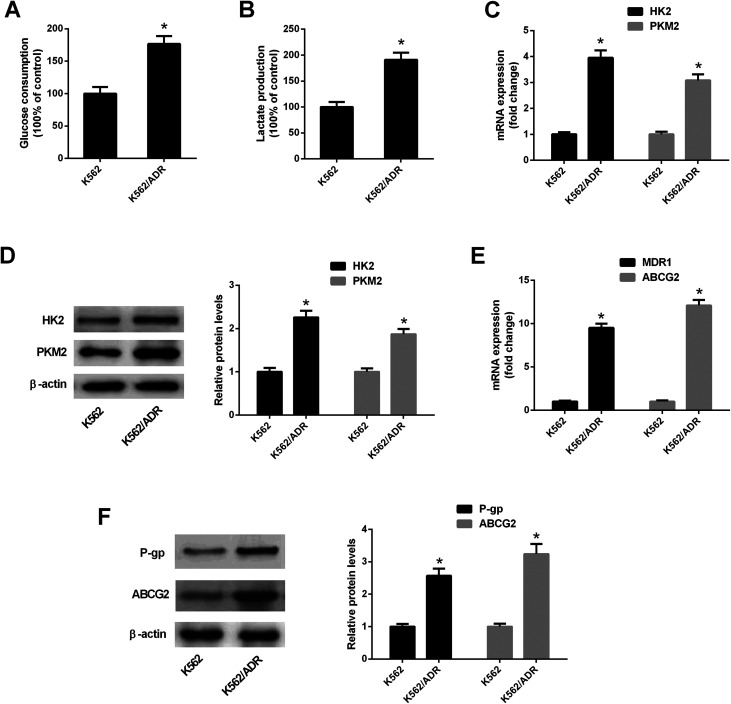
K562/ADR cells exhibited increased glycolytic activity and MDR gene expression. (A and B) Glucose consumption level and lactate amount was detected by matched commercial kits in K562 and K562/ADR cells. (C and E) RT-qPCR assay was carried out to measure mRNA levels of HK2, PKM2, MDR1 and ABCG2 in K562 and K562/ADR cells. (D and F) Protein levels of HK2, PKM2, P-gp and ABCG2 were determined by western blot assay in K562 and K562/ADR cells. *P < 0.05.

To explore the relationship of glycolysis and drug resistance, glucose consumption and lactate production levels were firstly detected in ADR-resistant K562 cells (K562/ADR) and its parental cells (K562). Results showed that K562/ADR cells consumed more glucose and produced more lactate compared with K562 cells (Fig. 1A and B), hinting that K562/ADR cells had higher glycolytic activity. To further validate this conclusion, expression of enzymes critical for glycolysis pathway was examined in K562/ADR and K562 cells. As presented in Fig. 1C and D, the mRNA and protein levels of HK2 and PKM2 (two key enzymes in the glycolysis pathway) were upregulated in K562/ADR cells relative to K562 cells. Also, K562/ADR cells presented elevated MDR1 mRNA and P-gp protein expression as well as increased ABCG2 mRNA and protein expression in comparison with K562 control cells (Fig. 1E and F). These data suggested that K562/ADR cells exhibited increased glycolytic activity and MDR gene expression.

### 3.2. ART enhanced the inhibitory effect of ADR on cell viability in K562/ADR cells

**Fig. 2:**
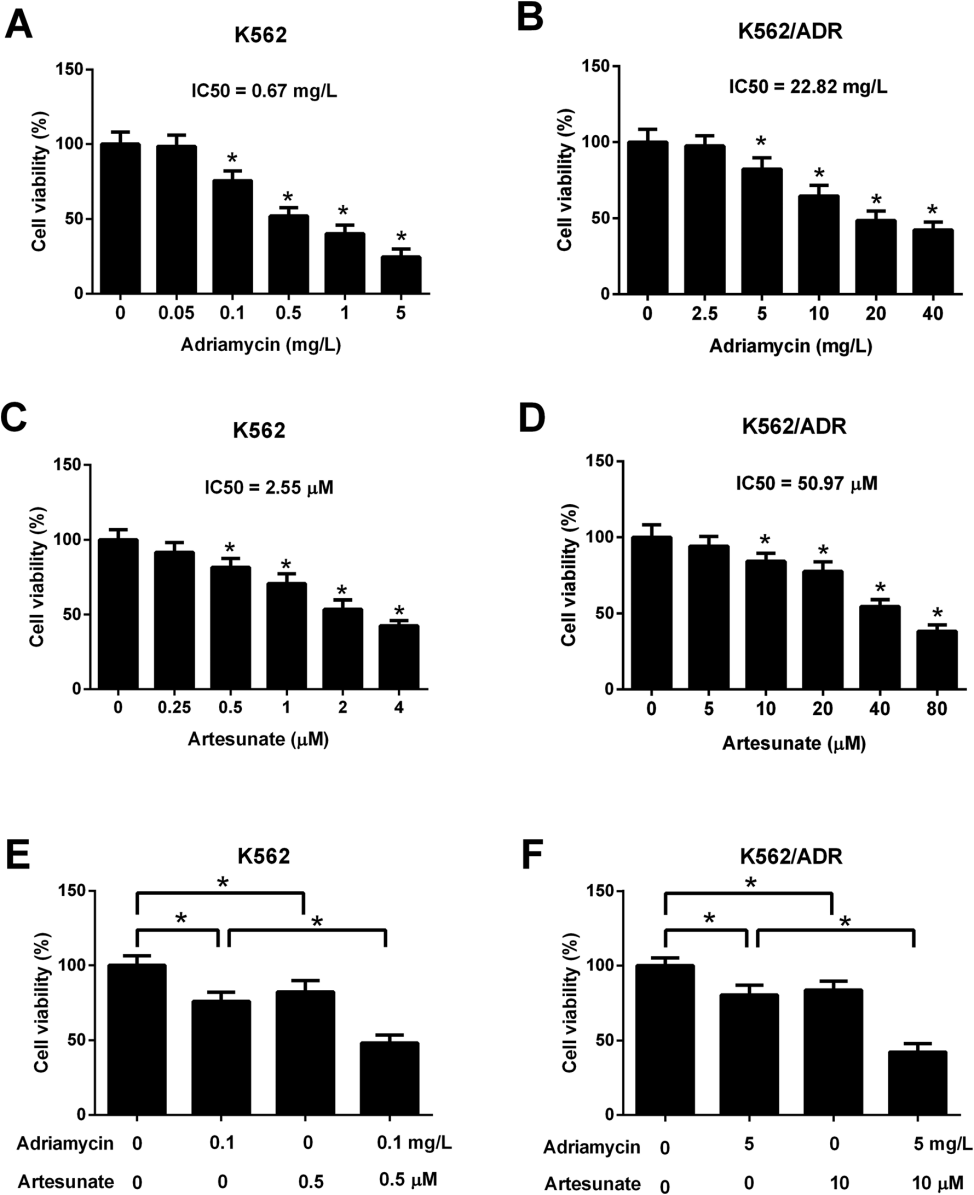
ART enhanced the inhibitory effect of ADR on the viability of K562/ADR cells. (A) K562 cells were stimulated with various doses (0, 0.05, 0.1, 0.5, and 1, 5 mg L^−1^) of ADR for 48 h. Cell viability was measured by CCK-8 assay. *P < 0.05 *vs.* untreated cells. (B) K562/ADR cells were stimulated with various doses (0, 2.5, 5, 10, 20, and 40 mg L^−1^) of ADR for 48 h. Cell viability was measured by CCK-8 assay. *P < 0.05 *vs.* untreated cells. (C) The viability of K562 cells was examined by CCK-8 assay at 48 h after the treatment of different concentrations of ART (0, 0.25, 0.5, 1, 2, and 4 μM). *P < 0.05 *vs.* untreated cells. (D) The viability of K562/ADR cells was examined by CCK-8 assay at 48 h after treatment of different concentrations of ART (0, 5, 10, 20, 40, and 80 μM). *P < 0.05 *vs.* untreated cells. (E) K562 cells were treated with 0.1 mg L^−1^ ADR, 0.5 μM ART, or 0.1 mg L^−1^ ADR + 0.5 μM ART, followed by the detection of cell viability at 48 h post treatment. *P < 0.05. (F) K562/ADR cells were treated with 5 mg L^−1^ ADR, 10 μM ART, or 5 mg L^−1^ ADR + 10 μM ART, followed by the detection of cell viability at 48 h post treatment. *P < 0.05.

Leukemia cells were treated with ART for 48 h in a previous study,^18^ therefore, an incubation time of 48 h was used in this study. CCK-8 assay showed that cell viability was gradually reduced in K562 cells treated with increasing doses of ADR (0.1–5 mg L^−1^) (Fig. 2A). ADR stimulation at the concentration range of 5–40 mg L^−1^ resulted in a reduction of K562/ADR cell viability in a dose-dependent manner (Fig. 2B). These findings suggested that K562/ADR cells displayed resistance to ADR *versus* K562 cells. Additionally, a dose-dependent downregulation of cell viability was observed in K562 and K562/ADR cells stimulated with different concentrations of ART (Fig. 2C and D). To clearly observe the effect of ART on ADR cytotoxicity, 0.1 mg L^−1^ of ADR and 0.5 μM of ART (for K562 cells) or 5 mg L^−1^ of ADR and 10 μM of ART (for K562/ADR cells) were selected for the following experiments. Further analysis showed that ART potentiated the inhibitory effect of ADR on the viability of K562 (Fig. 2E) and K562/ADR cells (Fig. 2F).

### 3.3. ART accelerated ADR-induced apoptosis in K562/ADR cells

**Fig. 3:**
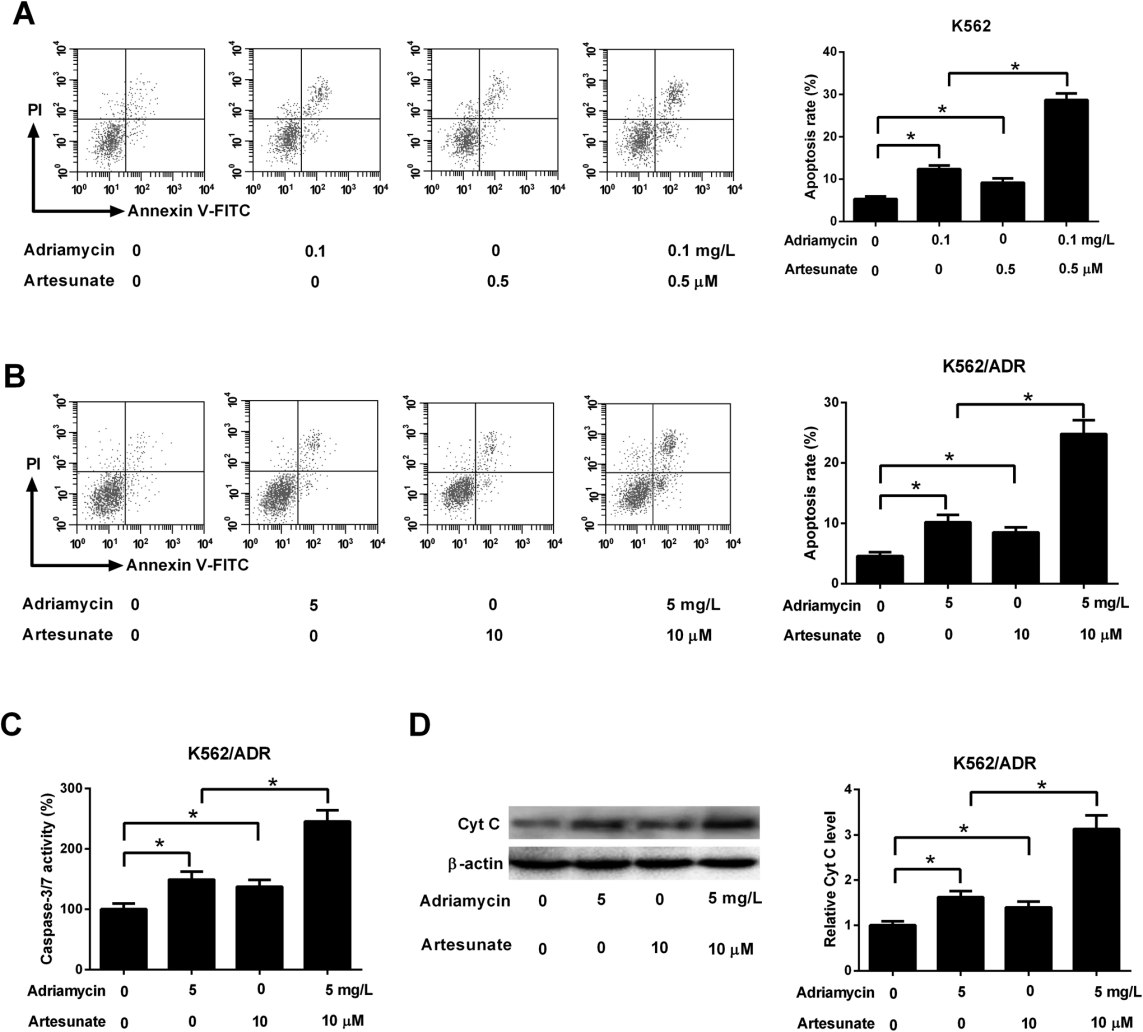
ART accelerated ADR-induced apoptosis in K562/ADR cells. (A) K562 cells were treated with 0.1 mg L^−1^ ADR, 0.5 μM ART, or 0.1 mg L^−1^ ADR + 0.5 μM ART, followed by the measurement of cell apoptotic rate. (B–D) K562/ADR cells were treated with 5 mg L^−1^ ADR, 10 μM ART, or 5 mg L^−1^ ADR + 10 μM ART, followed by the measurement of cell apoptotic rate, caspase-3/7 activity and Cyt *c* protein level at 48 h following stimulation. *P < 0.05. Untreated cells functioned as the negative control.

As shown in Fig. 3A, both ADR (0.1 mg L^−1^) and ART (0.5 μM) induced apoptosis of K562 cells, and apoptosis rate was increased after co-incubation with ADR and ART, suggesting ART enhanced ADR-induced apoptosis in K562 cells. We further demonstrated that the introduction of ADR (5 mg L^−1^) or ART (10 μM) resulted in an increase in apoptotic rate, caspase-3/7 activity and Cyt *c* protein level in K562/ADR cells (Fig. 3B–D), suggesting that ADR (5 mg L^−1^) or ART (10 μM) induced apoptosis of K562/ADR cells. Additionally, ART potentiated ADR-induced apoptosis in K562/ADR cells, as evidenced by the obvious upregulation of apoptotic rate, caspase-3/7 activity and Cyt *c* protein level in K562/ADR cells (Fig. 3B–D).

### 3.4. ART reversed ADR-induced MDR gene expression in K562/ADR cells

**Fig. 4:**
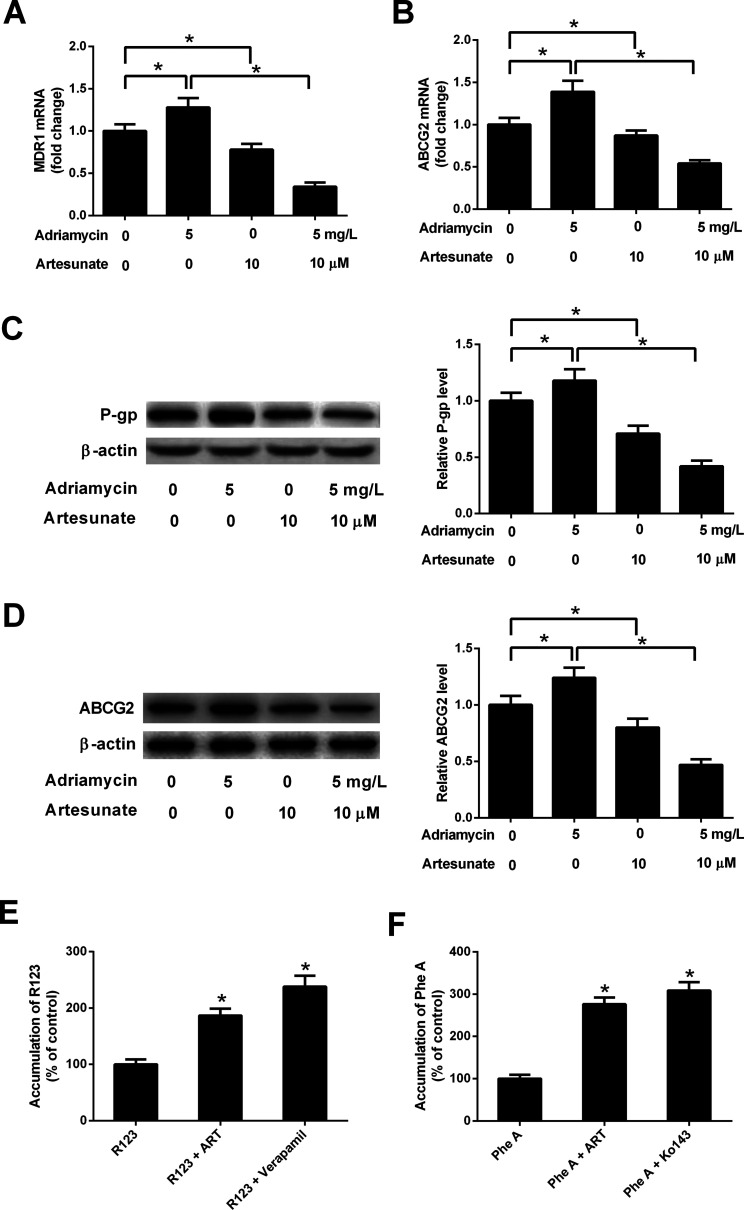
ART reversed ADR-induced MDR gene expression and P-gp and ABCG2 activities in K562/ADR cells. (A–D) K562/ADR cells were treated with 5 mg L^−1^ ADR, 10 μM ART, or 5 mg L^−1^ ADR + 10 μM ART for 48 h. MDR1 and ABCG2 mRNA levels were measured by RT-qPCR assay. Protein expression of P-gp and ABCG2 was determined by western blot assay. *P < 0.05. Untreated cells acted as the negative control. (E and F) K562/ADR cells were treated with 10 μM ART for 24 h, 50 μM verapamil (a positive control inhibitor of P-gp) for 0.5 h, or 10 μM Ko140 (a positive control inhibitor of ABCG2) for 0.5 h at 37 °C. Then, P-gp substrate rhodamine123 (R123; 5 μM) or ABCG2 substrate pheophorbide A (Phe A; 10 μM) was added and incubated for an additional 0.5 h. Intracellular accumulation of R123 or Phe A was evaluated by measurement of fluorescence intensity using a Microplate Reader. *P < 0.05. Cells treated with R123 or Phe A acted as the control group.

ADR (5 mg L^−1^) induced an increase in MDR1 mRNA, ABCG2 mRNA, P-gp protein and ABCG2 protein levels in K562/ADR cells (Fig. 4A–D). Conversely, MDR1 mRNA, ABCG2 mRNA, P-gp protein and ABCG2 protein expression was reduced in K562/ADR cells in response to ART (10 μM) stimulation (Fig. 4A–D). The introduction of ART reversed the promotive effect of ADR on *mdr1* and *abcg2* gene expression in K562/ADR cells (Fig. 4A–D). Furthermore, P-gp substrate R123 and ABCG2 substrate Phe A were applied to test the effect of ART on P-gp and ABCG2 activities. As shown in Fig. 4E, both ART and P-gp inhibitor verapamil increased intracellular accumulation of R123. Similarly, intracellular accumulation of Phe A was increased after treatment with ART or ABCG2 inhibitor Ko143 (Fig. 4F). These data suggested that ART reversed ADR-induced MDR gene expression and inhibited P-gp and ABCG2 activities in K562/ADR cells.

### 3.5. ART potentiated ADR-mediated inhibitory effect on glycolysis in K562/ADR cells

**Fig. 5:**
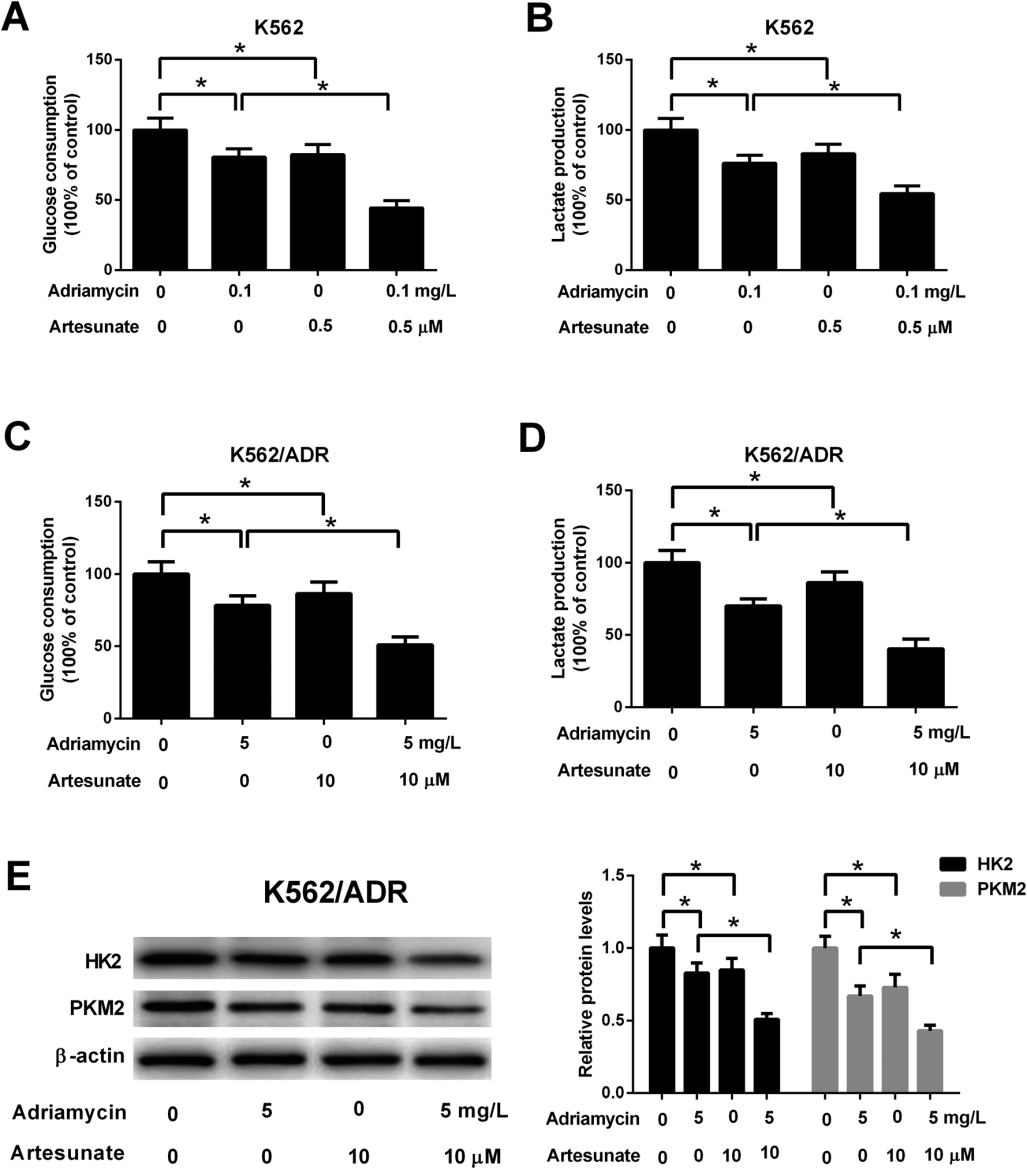
ART potentiated ADR-mediated inhibitory effect on glycolysis in K562/ADR cells. (A and B) K562 cells were treated with 0.1 mg L^−1^ ADR, 0.5 μM ART, or 0.1 mg L^−1^ ADR + 0.5 μM ART, followed by the determination of glucose consumption and lactate production. (C–E) K562/ADR cells were treated with 5 mg L^−1^ ADR, 10 μM ART, or 5 mg L^−1^ ADR + 10 μM ART for 48 h, followed by the determination of glucose consumption, lactate production, HK2 and PKM2 protein levels. *P < 0.05. Untreated cells acted as the negative control.

Glucose consumption and lactate production were decreased after treatment with ADR (0.1 mg L^−1^) or ART (0.5 μM). ART treatment potentiated ADR-mediated inhibitory effect on glycolysis in K562 cells (Fig. 5A and B). Further explorations showed that ADR (5 mg L^−1^) or ART (10 μM) treatment inhibited glycolysis in K562/ADR cells, as presented by the decreased glucose consumption, lactate production, HK2 protein level and PKM2 protein level in ADR- or ART-treated cells compared with untreated cells (Fig. 5C–E). The inhibitory effect of ADR on glycolysis was potentiated by ART in K562/ADR cells (Fig. 5C–E).

### 3.6. Inhibition of glycolysis resulted in reduced cell viability, increased apoptosis, reduced MDR gene expression in K562/ADR cells

**Fig. 6:**
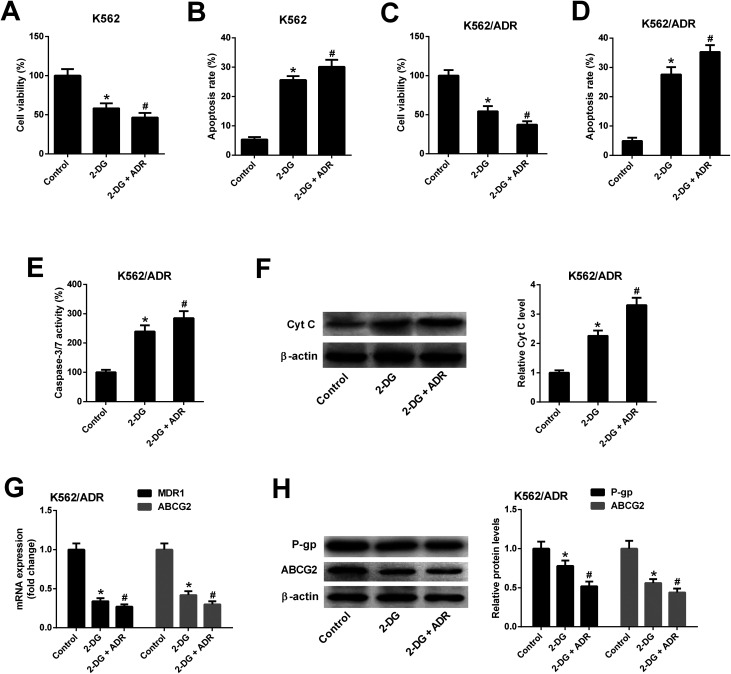
Inhibition of glycolysis resulted in reduced cell viability, increased apoptosis, reduced MDR gene expression in K562/ADR cells. K562 cells were treated with 2-DG (5 mM) or 2-DG (5 mM) plus ADR (0.1 mg L^−1^) for 48 h. Cell viability (A) and apoptosis rate (B) was determined by CCK-8 assay and flow cytometry, respectively. K562/ADR cells were treated with 2-DG (5 mM) or 2-DG (5 mM) plus ADR (5 mg L^−1^) for 48 h. Cell viability (C) was determined by CCK-8 assay. Cell apoptosis was assessed by detecting apoptotic rate (D), caspase-3/7 activity (E) and Cyt *c* protein level (F). MDR1 and ABCG2 mRNA levels (G) were detected by RT-qPCR assay. Protein levels of P-gp and ABCG2 (H) were measured by western blot assay. *P < 0.05 *vs.* control group. ^#^*P* < 0.05 *vs.* 2-DG treatment group.

To explore whether ART affected K562 cell viability and apoptosis by inhibiting glycolysis, the glycolysis inhibitor 2-DG was used. As shown in Fig. 6A and B, 2-DG inhibited cell viability and induced apoptosis in K562 cells. The effects of 2-DG were enhanced in 2-DG plus ADR group. To further demonstrate that ART affected K562/ADR cell viability and apoptosis by inhibiting glycolysis, the effect of 2-DG on cell viability, apoptosis, MDR gene expression was examined. Results showed that inhibition of glycolysis by 2-DG resulted in the reduction of cell viability (Fig. 6C) and the increase of apoptotic rate (Fig. 6D), caspase-3/7 activity (Fig. 6E) and Cyt *c* level (Fig. 6F) in K562/ADR cells. The effects of 2-DG were enhanced in 2-DG plus ADR group. The levels of MDR1 mRNA, ABCG2 mRNA, P-gp protein and ABCG2 protein were reduced in K562/ADR following the inhibition of glycolysis and the effects of 2-DG were enhanced in 2-DG plus ADR group (Fig. 6G and H).

## 4. Discussion

Recently, accumulating oncologists proposed that glycolysis could act as a target area to overcome chemoresistance for cancer patients.^22,23,27^ Moreover, mounting evidences show that drug resistance including MDR is closely correlated with the abnormal expression of transport proteins, anomalous tumor metabolism, and increased glycolysis and lactic acid production in cancers including leukemias.^28,29^ For example, the inhibition of glycolysis reduced the activity of Akt/mammalian target of rapamycin (mTOR)/hypoxia-inducible factor-1α (HIF-1α) axis and improved tamoxifen sensitivity in antiestrogen-resistant breast cancer cells.^30^ The suppression of glycolysis by 2-DG, lonidamine (LND), or 3-bromopyruvate (3-BrPA) enhanced the sensitivity of prednisolone-resistant ALL cells to glucocorticoids.^31^

Previous studies showed that ADR resistance was closely related with glycolysis responses in AML and CML.^32,33^ For instance, Song *et al.*^32^ showed that the expression of glucose metabolism-related genes such as *glucose transporter 1* (*GLUT1*) and *HK2* was markedly upregulated in blasts from AML patients after chemotherapy with no remission compared with healthy control group and patients with complete remission or partial remission, indicating that the relevance between of glucose metabolism and chemotherapy responses in AML patients. Glucose consumption and HK2 expression was increased in ADR-resistant HL-60/ADR cells compared with ADR-sensitive HL-60 cells (AML cells), further hinting the link of glucose metabolism and ADR resistance in AML cells. Inhibition of glycolysis by 2-DG or 3-BrPA further enhanced the cytotoxicity of ADR in HL-60 and HL-60/ADR cells. Zhang *et al.*^33^ revealed that glycolytic activity was markedly upregulated in K562/ADR cells *versus* K562 cells and the inhibition of glycolysis restored the sensitivity of K562/ADR cells to ADR. The phosphatidylinositol-3-kinase (PI3K)/AKT pathway was activated in K562/ADM cells, which might account for the enhancement of glycolysis and drug resistance.^34^ Glycolytic inhibition causes a decrease in energy supply, resulting in suppression of the ATP-dependent drug-efflux activities of P-gp, which may represent a strategy to overcome multidrug resistance.^33^ Consistently, our study also confirmed that K562/ADR cells had stronger glycolytic activity relative to K562 cells. Prior comparative proteomics analysis showed that the expression of glycolysis-related enzymes such as enolase and aldolase was markedly upregulated in K562/ADR cells compared with K562 cells.^35^ In accordance with the earlier report,^36^ a marked increase of *mdr1* and *abcg2* gene expression at mRNA and protein levels was observed in K562/ADR cells relative to K562 cells. Similarly, Liu *et al*.^37^ pointed out that *abcg2* gene expression was markedly upregulated in ADR-resistant esophageal cancer cells. In a word, these data hinted the possible correlation between MDR and glycolysis.

As reported in previous documents, ART suppressed cell proliferation and induced cell apoptosis in K562 CML cells^18,38^ and hampered the growth of human CML xenograft tumors *in vivo*.^19^ Kumar *et al.*^16^ pointed out that ART augmented the cytotoxicity of ADR or cytarabine in human AML cells and primary CD34+ patient blasts. Kim *et al.*^19^ further showed that ART enhanced the cytotoxicity of ADR, paclitaxel and docetaxel in KBM-5 CML cells. Our study aimed to further investigate whether ART could augment the cytotoxicity of ADR by regulating glycolysis in K562/ADR cells. Results showed that ART could enhance the cytotoxicity of ADR, as evidenced by reduced cell viability and increased apoptotic activity in ADR-treated K562/ADR cells following the stimulation of ART.

Previous studies showed tumor cell lines overexpressing MDR1, multidrug resistance protein 1 (MRP1) and ABCG2 were not cross-resistant to ART,^39–41^ indicating that ART is not a substrate for MDR1, MRP1 and ABCG2. In the subsequent experiments, the effect of ART on expression and function of MDR1 and ABCG2 was investigated. Results showed that ART downregulated *mdr1* and *abcg2* gene expression and reversed ADR-induced *mdr1* and *abcg2* gene expression in K562/ADR cells. Similarly, previous studies showed that ART reversed the ADR resistance by reducing ABCG2 expression in esophageal cancer *in vitro* and *in vivo*.^21,42^ However, Reungpatthanaphong *et al.*^43^ showed that ART poorly suppressed P-gp function, but enhanced cytotoxicity of ADR in K562/ADR cells. Also, Sertel *et al.*^44^ pointed out that ART had no effect on the function of P-gp and ABCG2 in ADR-resistant leukemia cell line P388/dx and MDCKII cells. The effect of ART on the function of P-gp and ABCG2 need to be further explored.

HK2 and PKM2 are two key enzymes in the glycolysis pathway.^45^ It has been demonstrated that HK2 and PKM2 are involved in drug resistance in cancer.^46^ High level HK2 is associated with resistance to rituximab and chemotherapy agents in aggressive lymphoma.^47^ A recent study showed that overexpression of miR-202 reversed imatinib resistance in imatinib-resistant chronic myeloid leukemia cells through glycolysis inhibition by targeting HK2.^48^ HK2 also has been reported to promote cisplatin resistance in drug-resistant human ovarian cancer cells by enhancing autophagy.^49^ Shi *et al.*^50^ found that silencing of PKM2 enhanced antitumor activity of docetaxel in human lung cancer cells and in A549 cell xenografts, suggesting that targeting tumor glycolysis can increase the efficacy of chemotherapy. He *et al.*^51^ showed that overexpression of miR-122 in 5-fluorouracil-resistant colon cancer cells resensitized 5-fluorouracil resistance through the inhibition of PKM2 both *in vitro* and *in vivo*, revealing that glycolysis inhibition might be a promising therapeutic strategy to overcome 5-fluorouracil resistance. In the current study, we demonstrated that ADR or ART stimulation inhibited glycolysis in K562/ADR cells. ART strengthened ADR-induced glycolysis inhibition in K562/ADR cells. Moreover, inhibition of glycolysis by 2-DG resulted in reduced cell viability, increased apoptosis, and downregulated *mdr1* and *abcg2* gene expression in K562/ADR cells, validating that glycolysis could influence cell viability, apoptosis and MDR gene expression in ADR-resistant K562 cells.

## 5. Conclusion

Collectively, our data showed that ART potentiated ADR cytotoxicity by inhibiting glycolysis and downregulating *mdr1* and *abcg2* gene expression in ADR-resistant K562 cells, indicating the values of ART and ADR combined therapy in reducing drug resistance in leukemia and further elucidating the crucial roles of glycolysis in drug resistance. However, *in vivo* experiments need to be conducted to further validate the vital values of ART in the mitigation of ADR resistance. Moreover, the effect and molecular basis of ART on ADR resistance also requires to be further investigated in other leukemia cell lines. Additionally, it is still poor elaborated whether ART could enhance the cytotoxicity of leukemia cells to other chemotherapeutic drugs.

## Conflicts of interest

The authors declare that they have no conflict of interest.

## Supplementary Material
